# ADDRESSING BENZODIAZEPINE PRESCRIBING PRACTICES AMONGST ELDERLY PTSD VETERANS DURING THE COVID-19 PANDEMIC

**DOI:** 10.1093/geroni/igad104.2261

**Published:** 2023-12-21

**Authors:** Deanna Fernandes, Josepha Cheong

**Affiliations:** University of Florida, Gainesville, Florida, United States; North Florida/South Georgia Veterans Affairs Medical Center, Gainesville, Florida, United States

## Abstract

Psychotropic medication prescribing amongst elderly veterans has increased over the course of the COVID-19 pandemic despite the fact that psychotropics such as benzodiazepines, when being prescribed for a period over 2 weeks, are considered as relatively contraindicated amongst such patients with an additional Post-Traumatic Stress Disorder (PTSD) diagnosis due to a worsening of disease state symptoms. The objectives of my study are to inform clinicians and healthcare providers regarding the true extent of current benzodiazepine prescribing practices amongst elderly Veterans with PTSD and to provide a clinical aid/tool for education and improvement of such practices within the setting of the Veterans Affairs Medical Center. A one-page summarized aid will be drafted based upon provider feedback as well as from current Pharmacy Benefits Management Network resources to improve clinical practices with regards to the topics of benzodiazepine recommended length of use, risks of use amongst elderly Veterans, and risks amongst PTSD populations. While benzodiazepine prescribing practices have increased recently during the time of the COVID-10 pandemic, clinical tools may be drafted to improve such practices. Further studies are needed to determine the true impacts of such a tool on the national level as well as upon direct patient care.

